# Rectal dose-sparing effect with bioabsorbable spacer placement in carbon ion radiotherapy for sacral chordoma: dosimetric comparison of a simulation study

**DOI:** 10.1093/jrr/rrab013

**Published:** 2021-03-30

**Authors:** Shintaro Shiba, Masahiko Okamoto, Mutsumi Tashiro, Hiroomi Ogawa, Katsuya Osone, Takashi Yanagawa, Isaku Kohama, Shohei Okazaki, Yuhei Miyasaka, Naoto Osu, Hirotaka Chikuda, Hiroshi Saeki, Tatsuya Ohno

**Affiliations:** Department of Radiation Oncology, Gunma University Graduate School of Medicine, 3-39-22 Showa-machi, Maebashi, Gunma, Japan; Gunma University Heavy Ion Medical Center, 3-39-22 Showa-machi, Maebashi, Gunma, Japan; Department of Radiation Oncology, Gunma University Graduate School of Medicine, 3-39-22 Showa-machi, Maebashi, Gunma, Japan; Gunma University Heavy Ion Medical Center, 3-39-22 Showa-machi, Maebashi, Gunma, Japan; Department of General Surgical Science, Gunma University Graduate School of Medicine, 3-39-22 Showa-machi, Maebashi, Gunma, Japan; Department of General Surgical Science, Gunma University Graduate School of Medicine, 3-39-22 Showa-machi, Maebashi, Gunma, Japan; Department of Orthopedic Surgery, Gunma University Graduate School of Medicine, 3-39-22 Showa, Maebashi, Gunma, Japan; Department of Orthopedic Surgery, Gunma Prefectural Cancer Center, 617-1 Takahayashinishi-cho, Ota, Gunma, Japan; Department of Orthopedic Surgery, Gunma University Graduate School of Medicine, 3-39-22 Showa, Maebashi, Gunma, Japan; Gunma University Heavy Ion Medical Center, 3-39-22 Showa-machi, Maebashi, Gunma, Japan; Gunma University Heavy Ion Medical Center, 3-39-22 Showa-machi, Maebashi, Gunma, Japan; Department of Radiation Oncology, Gunma University Graduate School of Medicine, 3-39-22 Showa-machi, Maebashi, Gunma, Japan; Department of Orthopedic Surgery, Gunma University Graduate School of Medicine, 3-39-22 Showa, Maebashi, Gunma, Japan; Department of General Surgical Science, Gunma University Graduate School of Medicine, 3-39-22 Showa-machi, Maebashi, Gunma, Japan; Department of Radiation Oncology, Gunma University Graduate School of Medicine, 3-39-22 Showa-machi, Maebashi, Gunma, Japan

**Keywords:** carbon ion radiotherapy, bioabsorbable spacer, spacer placement, virtual endoscopy, sacral chordoma

## Abstract

It is difficult to treat patients with an inoperable sarcoma adjacent to the gastrointestinal (GI) tract using carbon ion radiotherapy (C-ion RT), owing to the possible development of serious GI toxicities. In such cases, spacer placement may be useful in physically separating the tumor and the GI tract. We aimed to evaluate the usefulness of spacer placement by conducting a simulation study of dosimetric comparison in a patient with sacral chordoma adjacent to the rectum treated with C-ion RT. The sacral chordoma was located in the third to fourth sacral spinal segments, in extensive contact with and compressing the rectum. Conventional C-ion RT was not indicated because the rectal dose would exceed the tolerance dose. Because we chose spacer placement surgery to physically separate the tumor and the rectum before C-ion RT, bioabsorbable spacer sheets were inserted by open surgery. After spacer placement, 67.2 Gy [relative biological effectiveness (RBE)] of C-ion RT was administered. The thickness of the spacer was stable at 13–14 mm during C-ion RT. Comparing the dose–volume histogram (DVH) parameters, D_max_ for the rectum was reduced from 67 Gy (RBE) in the no spacer plan (simulation plan) to 45 Gy (RBE) in the spacer placement plan (actual plan) when a prescribed dose was administered to the tumor. Spacer placement was advantageous for irradiating the tumor and the rectum, demonstrated using the DVH parameter analysis.

## INTRODUCTION

Carbon ion radiotherapy (C-ion RT) has been used to treat various types of cancer [1–5]. C-ion RT has also been performed for bone and soft tissue sarcomas, with clinical results comparable with those of surgery [4–6]. Imai *et al*. reported that the administration of C-ion RT for sacral chordoma demonstrated a 5-year overall survival and local control rates of 81% and 77%, respectively, with tolerable toxicities and no bladder and bowel dysfunctions [4]. Therefore, C-ion RT for sacral chordoma is a highly curative and minimally invasive treatment.

**Fig. 1.**
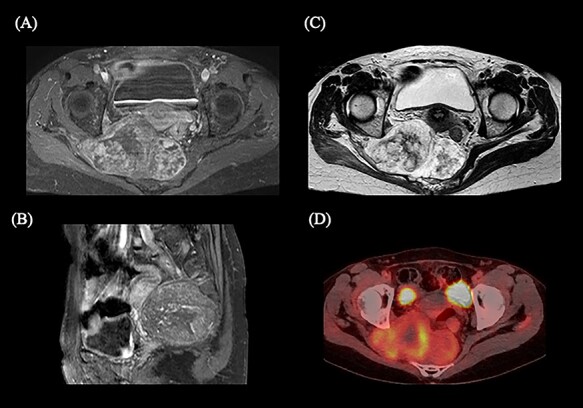
Magnetic resonance imaging (MRI) and 2-deoxy-2-[^18^F]fluoro-d-glucose (FDG)-positron emission tomography (PET)/computed tomography (CT) showing the sacral chordoma before spacer placement and the initiation of carbon ion radiotherapy. (A) Axial MRI of the gadolinium-enhanced T1-weighted image. (B) Sagittal MRI of the gadolinium-enhanced T1-weighted image. (C) Axial MRI of the T2-weighted axial image. (D) Axial FDG-PET/CT.

However, C-ion RT has limited indications. The risk of serious toxicities such as gastrointestinal (GI) perforation may increase in tumors located adjacent to the GI tract. Therefore, the marginal dose to such tumors should be reduced to prevent severe toxicities; additionally, radical doses should not be administered. The usefulness of spacer placement, which physically separates the tumor and the GI tract with Gore-Tex sheets (W.L. Gore and Associates, Newark, DE, USA) or bioabsorbable polyglycolic acid (PGA) sheets, in overcoming this limitation has been reported [4, 7–10]. Imai *et al*. reported that 5% of patients with sacral chordoma required spacer placement. To date, only a limited number of reports on the usefulness and safety of spacer placement are available. In addition, these studies have not fully analyzed the advantages of spacer placement in C-ion RT by comparing dose–volume histogram (DVH) parameters, and there is only a single published report regarding a bioabsorbable PGA spacer [10].

In the present study, we investigated the usefulness of a spacer to evaluate GI doses by dosimetric comparison with and without spacer placement in a patient with sacral chordoma. Additionally, we visualized dose distributions in the rectal wall using virtual endoscopy.

## MATERIALS AND METHODS

### Patient

A 70-year-old Japanese woman with sacral chordoma was referred to the Gunma University Heavy Ion Medical Center for C-ion RT.

**Fig. 2.**
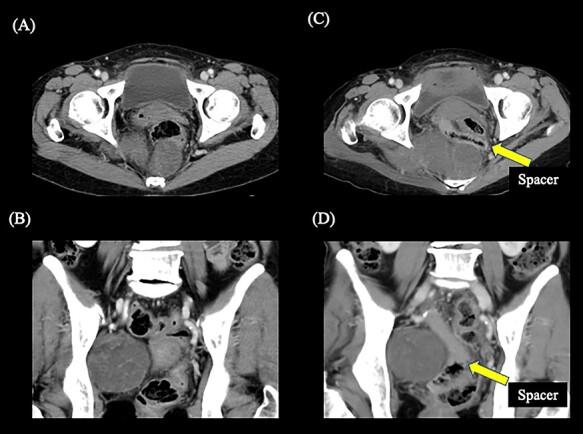
Contrast-enhanced computed tomography showing the positional relationship between the tumor and the rectum. (A) Axial image before spacer placement. (B) Coronal image before spacer placement. (C) Axial image after spacer placement. (D) Coronal image after spacer placement. The yellow arrow shows the spacer. The gas is formed within the spacer due to the process of hydrolysis after placement of the spacer.

Histological analysis of the biopsy specimen of the patient revealed a chordoma. Magnetic resonance imaging detected a tumor (124 × 76 × 67 mm) located in the third to fourth sacral spinal segment with good contrast in gadolinium-enhanced T1-weighted images and increased intensity on T2-weighted images extending to the right piriformis muscle (Fig. 1A–C). The tumor was located in extensive contact with the rectum, and the rectum was compressed by the tumor (Fig. 2A, B; Supplementary Fig. 1). 2-Deoxy-2-[^18^F]fluoro-d-glucose (FDG)-positron emission tomography (PET)/computed tomography (CT) showed abnormal FDG uptake in the sacral tumor (Fig. 1D). FDG-PET/CT and contrast-enhanced CT showed no evidence of metastasis to lymph nodes or distant organ sites. The patient was diagnosed with stage IB chordoma based on the eighth edition of the Union for International Cancer Control/American Joint Committee on Cancer TNM staging system [11].

**Fig. 3.**
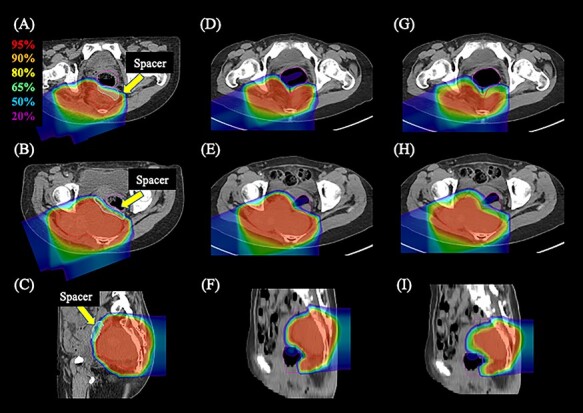
Dose distribution of carbon ion radiotherapy. (A–C) Axial and sagittal images of the actual plan with spacer placement. (D–F) Axial and sagittal images of the simulated plan without spacer placement that prioritized the dose coverage to the gross tumor volume (GTV). (G–I) Axial and sagittal images of the simulated plan without spacer placement that prioritized dose restrictions to the rectum. The area within the red outline is the GTV, the light green is the spacer and the magenta is the rectum. Highlighted are 95% (red), 90% (orange), 80% (yellow), 65% (green), 50% (blue) and 20% (purple) isodose curves [100% was 67.2 Gy (RBE)].

Conventional C-ion RT was not indicated initially because the adjacent rectal dose was expected to exceed the tolerance dose. The treatment strategy was discussed by the cancer board of the hospital, and spacer placement surgery before C-ion RT was chosen as a candidate treatment approach. The study protocol was approved by the ethics committee of Gunma University Graduate School of Medicine and informed consent was obtained from the patient before the initiation of therapy. The patient successfully underwent open surgery for the placement of the bioabsorbable 1 cm PGA spacer sheets between the tumor and the rectum (Fig. 2C, D; Supplementary Fig. 2). Six weeks after the spacer placement surgery, C-ion RT was initiated. The administered dose of C-ion RT was 67.2 Gy relative biological effectiveness (RBE) in 16 fractions for 4 weeks. Figure 3A–C shows the dose distribution of C-ion RT.

### Dose constrains and DVH parameter analysis

We compared DVH parameters in the gross tumor volume (GTV) and rectum in three treatment plans: actual plan (with spacer); simulated plan prioritizing dose coverage to the GTV (without spacer) (sim-plan GTV); and simulated plan prioritizing dose restrictions to the rectum (without spacer) (sim-plan Rec). The simulated plans were calculated using diagnostic CT before the spacer placement surgery. The dose constraints in the actual plan (with spacer) were the maximum dose (D_max_) <60 Gy (RBE) and the dose to 2 cm^3^ (D_2cc_) <45 Gy (RBE) administered to the rectum, and the percentage of the GTV that received at least 95% of the administered dose (GTV V_95_) >99%; those in sim-plan GTV were GTV V_95_ >99%; and those in sim-plan Rec were D_max_ <60 Gy (RBE) and D_2cc_ <45 Gy (RBE) administered to the rectum [1, 12–15]. Next, we assessed the percentage of the minimum dose that covered 95% and 98% of the GTV (D_95_ and D_98_, respectively), D_max_, D_2cc_ and D_1cc_ of the rectum. Additionally, we visualized the dose distribution in the rectal wall using virtual endoscopy (AZE, Tokyo, Japan) for the actual plan and the sim-plan GTV.

## RESULTS

### Clinical outcomes

The patient completed C-ion RT as scheduled and only developed grade 1 acute radiation dermatitis (the Common Terminology Criteria for Adverse Effects version 4.0). Repeated CT revealed that the thickness of the bioabsorbable spacer sheets was stable between 13 and 14 mm during C-ion RT. The patient survived with no evidence of local recurrence or distant metastasis for 4 months. The thickness of the bioabsorbable PGA spacer was reduced to 3 mm at 4 months after the initiation of C-ion RT.

### Comparison of DVH parameters

Figure 3 shows the dose distribution of C-ion RT in the actual plan (Fig. 3A–C), sim-plan GTV (Fig. 3D–F) and sim-plan Rec (Fig. 3G–I). Table 1 shows the results of the DVH parameter analysis in the actual plan, sim-plan GTV and sim-plan Rec. The rectal dose parameter was reduced in the actual plan compared with that in the sim-plan GTV. The DVH parameters of the actual plan showed that the dose restriction of the rectum was maintained while maintaining the dose coverage of the GTV.

**Table 1:** Dose–volume histogram parameters

(A) Evaluation of parameters for the GTV			
	D_95_	D_98_	
The actual plan with spacer placement	100%	100%	
Simulated plan without spacer placement that prioritizes the dose coverage to the GTV	98.0%	96.8%	
Simulated plan without spacer placement that prioritizes dose restrictions to the rectum	85.5%	82.7%	
(B) Evaluation of parameters for the rectum.			
	D_max_ [Gy (RBE)]	D_1cc_ [Gy (RBE)]	D_2cc_ [Gy (RBE)]
The actual plan with spacer placement	41.2	16.7	14.6
Simulated plan without spacer placement that prioritizes the dose coverage to the GTV	67.6	55.5	51.0
Simulated plan without spacer placement that prioritizes dose restrictions to the rectum	55.4	42.8	40.0

GTV = gross tumor volume; D_95_ and D_98_ = the percentage of minimum dose that covered 95% and 98%, respectively.

D_max_, the maximum dose of the rectum; D_1cc_ and D_2cc_, the minimum doses of the most exposed small volumes in 1 and 2 cm^3^ of the rectum, and the maximum dose of the rectum (D_max_).

### Analysis of dose distribution in the rectal wall using virtual endoscopy

**Fig. 4.**
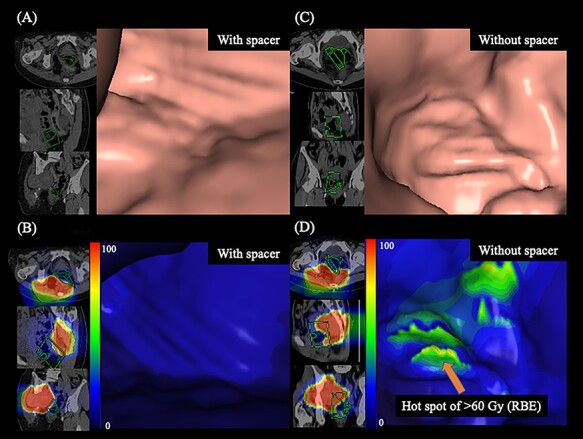
Observation of the rectal wall using virtual endoscopy. (A) Without dose distribution on planning CT. (B) Dose distribution with spacer placement (the actual plan). (C) Without dose distribution on diagnostic CT. (D) Dose distribution without spacer placement (simulated plan that prioritized the dose coverage to the gross tumor volume). Highlighted are 100% (red), 90% (orange), 75% (yellow), 50% (green) and <20% (blue) isodose curves [100% was 67.2 Gy (RBE)]. The orange arrow shows the higher dose spot of exceeding dose constraint of the rectal wall. The four-sided pyramid indicates the viewing direction, with the green square being nearest to the image.

Visualization of dose distribution using virtual endoscopy revealed the anatomical information of the rectal wall. Figure 4A shows virtual endoscopy images of planning CT with no dose distribution, while Figure 4B shows the images of dose distribution in the actual plan. Figure 4C shows virtual endoscopy images of diagnostic CT before spacer placement with no dose distribution, and Figure 4D shows the images of dose distribution in the sim-plan GTV. Figure 4A–D captured almost the same points on the rectal wall. Figure 4B shows no higher dose spot in the rectal wall, whereas Figure 4D shows a higher dose spot of exceeding dose constraint with 60 Gy (RBE) in the rectal wall.

## DISCUSSION

In the present study, we showed the usefulness of spacer placement in combination with C-ion RT through dosimetric comparison in a patient with sacral chordoma whose tumor was in extensive contact with the rectum. Comparison of DVH parameters showed clinically useful dose improvements in the target tumor and the rectum after spacer placement. This result suggested that spacer placement could expand patient indication, improve curability and reduce toxicities associated with the irradiation of tumors located close to the GI tract of patients.

Although C-ion RT has a higher dose localization property, the size of the penumbra showed that the distance from the position of the 80% dose level to that of the 20% level was ~2–3 mm in 4–12 cm of spread-out Bragg peak in the water phantom [16]. Therefore, when the tumor and the GI tract have a wide area of contact, it becomes difficult to administer a sufficient dose to the tumor while complying with dose constraints for the GI tract even by C-ion RT. Therefore, spacer placement is beneficial in treatment because the spacer displaces the GI tract from the tumor and enables the reduction of C-ion RT dose to the rectum. In the present case, using 1 cm spacer placement, we were able to radically treat the patient who was otherwise difficult to treat. However, attention must be paid to the indication of spacer placement. The duration from the decision of the treatment strategy to the initiation of C-ion RT after spacer placement might be 1–2 months. During this waiting period, there may be tumor growth and disease stage progression. Therefore, it is important to note that there is the possibility of missing the timing of local treatment, especially in rapidly growing tumors.

It has been reported that the tolerable dose to the rectum is 60 Gy (RBE) [12, 15], and the curative dose to the chordoma is 67.2 Gy (RBE) using C-ion RT. In the case of tumors located adjacent to the GI tract, high-dose irradiation to the GI tract cannot be avoided to maintain the tumor dose coverage. Moreover, full-dose irradiation (curative dose) for sacral chordoma cannot be tolerated by the rectum. Therefore, in cases where the tumor is located adjacent to the GI tract without spacer placement, the dose constraints for the rectum have priority over dose administration to the GTV for safer treatment. Therefore, it can be inferred that the tumor control rate may be decreased. In this report, using spacer placement, D_max_ for the rectum was reduced from 67 Gy (RBE) to 45 Gy (RBE), and a curative dose could be administered to the GTV. These results suggest that spacer placement was effective in this patient.

To date, there have been a few reports on the effectiveness and safety of spacer placement [6–8]. However, there is a risk of infection due to leaving the Gore-Tex sheets spacer after the C-ion RT [7]. In this report, we used bioabsorbable PGA sheets that were absorbed ~30 weeks after insertion, which may reduce the risk of infection. In a previous phase I study on particle therapy with PGA spacer placement, no patient developed an infection after spacer placement surgery; although the sample size was small, the study suggested that using a bioabsorbable PGA spacer may be safer than using a Gore-Tex sheets [10]. The use of an absorbable spacer has the advantage of reducing the risk of infection; however, it also has the disadvantage of reduced dose distribution due to the absorption by the spacer during treatment. Therefore, it is necessary to consider the change in thickness of the bioabsorbable spacer and, if a reduction in dose distribution occurs, replanning of C-ion RT will be required.

Additionally, we have demonstrated the merged dose distribution in the rectal wall using virtual endoscopy. In this report, a virtual endoscope was used to visually compare the C-ion RT dose of the rectal wall before and after the spacer placement. However, this attempt to evaluate dose distribution using a virtual endoscope has a limitation. Reconstruction of images obtained using a virtual endoscope needs the presence of rectal gas; however, this condition is unfavorable in the actual treatment, because rectal gas should be eliminated to reduce uncertainty in beam range in C-ion RT. To perform virtual endoscopy, it is necessary to acquire a CT scan for diagnosis in the presence of rectal gas, which is different from the treatment planning that should be done without rectal gas; and the dose distribution in a virtual endoscope was different from that in an actual plan. Therefore, accurate evaluation of dose distribution using a virtual endoscope is challenging, and it might be difficult to use this technique routinely. Nevertheless, we expect that virtual endoscopy could be used as a reference for evaluation of toxicities as well as defining whether they might have resulted from C-ion RT.

In conclusion, we reported a case of a patient with sacral chordoma treated using C-ion RT for whom the advantages of C-ion dose for the rectum was improved with spacer placement, and the superiority of spacer placement was clearly indicated by comparing DVH parameters. Spacer placement may provide favorable clinical outcomes in cases of tumors located adjacent to the GI tract. Additionally, we visualized dose distribution in the rectal wall to gain anatomical information using virtual endoscopy.

## Supplementary Material

Sup_Figure1_rrab013

Sup_Figure2_rrab013
