# Supplementary figures and images for: Rectal dose-sparing effect with bioabsorbable spacer placement in carbon ion radiotherapy for sacral chordoma: dosimetric comparison of a simulation study
Source: J Radiat Res. 2021 Mar 30;62(3):549–55. doi: 10.1093/jrr/rrab013 (PMC8127650; doi:10.1093/jrr/rrab013)

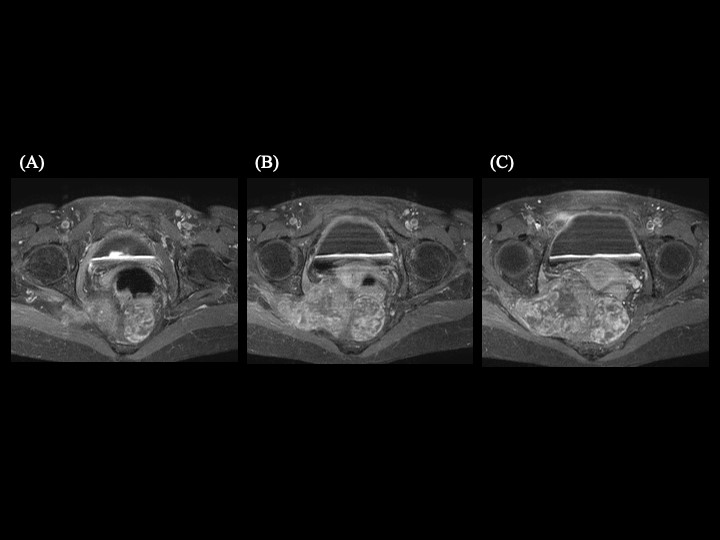

Supplement: Sup_Figure1_rrab013 [file sup_figure1_rrab013.jpeg]

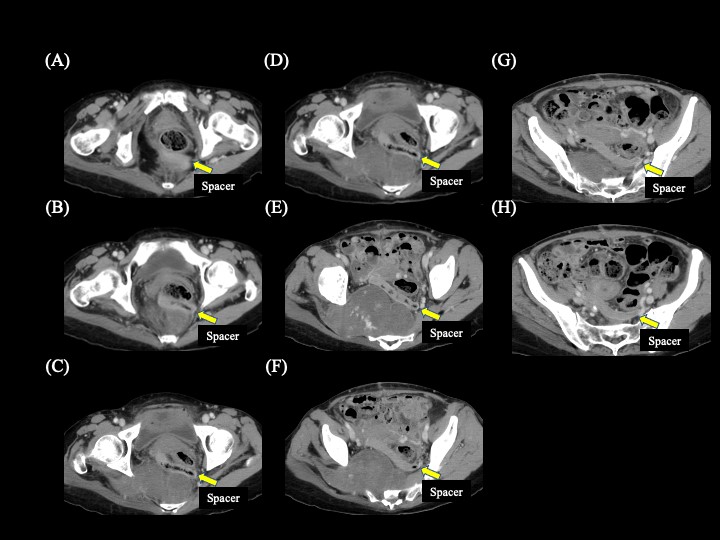

Supplement: Sup_Figure2_rrab013 [file sup_figure2_rrab013.jpeg]
